# Reuterin Isolated from *Lactobacillus reuteri* Indonesian Strain Affected Interleukin-8 and Human Beta Defensin-2 on Pathogens Induced-HaCat Cells

**DOI:** 10.21315/tlsr2022.33.2.5

**Published:** 2022-07-15

**Authors:** Armelia Sari Widyarman, Boy Muchlis Bachtiar, Endang Winiati Bahctiar

**Affiliations:** 1 Department of Microbiology, Faculty of Dentistry, Trisakti University, Kyai Tapa St. No. 260, Grogol, West Jakarta 11440, Indonesia; 2 Department of Oral Biology, Faculty of Dentistry, University of Indonesia, Salemba Raya St. No. 4, Salemba, Central Jakarta 10430, Indonesia

**Keywords:** Anti-Inflammatory, Human Beta Defensin-2, Interleukin-8 Reuterin, *Lactobacillus reuteri*

## Abstract

Probiotic *Lactobacillus reuteri* has positive effects on health through inhibiting pathogenic bacteria and the ability to reduce inflammation. This study investigates the ability of reuterin isolated from *L. reuteri* Indonesian strain for increasing mRNA expression of interleukin (IL)-8 and human beta-defensin (hBD)-2 gene by epithelial cells, after exposure to oral bacteria. *L. reuteri* isolated from Indonesian’s saliva, and species was confirmed by PCR, using 16S rRNA specific gene. To produce reuterin, the isolate was mixed in glycerol-containing MRS broth. Reuterin molecule’s weight was counted by SDS-PAGE. *Streptococcus mutans* ATCC-25175 and *Porphyromonas gingivalis* ATCC-33277 were put in water (80°C) for 30 min, and each killed bacterial (10^7^ CFU/mL) was inoculated into HaCat cell line (10^5^ cell/mL). Reuterin was added in different concentrations (100%, 50%, 25%, 12,5%) and different incubation time at 37°C, 5% CO_2_. RNA was extracted, and a reverse transcription procedure was performed to obtain cDNA. Subsequently, a quantitative PCR method was performed to analyse the transcription level of IL-8 and HBD-2 mRNA expressed by inflamed HaCat cells. All results were statistically analysed by ANOVA test. PCR assays showed that clinical isolates were *L. reuteri*. Quantitative PCR results showed reuterin decreased the expression of IL-8 and increased the expression of hBD-2 in all concentrations and time periods set in this study (*p <* 0.05). Reuterin isolated from *L. reuteri* Indonesian strain increased expression of human beta defensin-2 as antimicrobial peptide and may be useful in combating inflammation.

**Highlights**

- We have successfully proven the efficacy of reuterin from *L. reuteri* Indonesian strain (BEA 230424) in modulating inflammatory responses in HaCat cells that were stressed with *S. mutans* and *P. gingivalis*, by reducing the gene expression of pro-inflammatory interleukin-8.
- We have successfully proven the efficacy of reuterin from *L. reuteri* Indonesian strain (BEA 230424) in modulating inflammatory responses in HaCat cells that were stressed with *S. mutans* and *P. gingivalis*, by increasing the gene expression of innate defence peptide human beta defensin-2.
- We have shown that reuterin from *L. reuteri* Indonesian strain (BEA 230424) is not toxic for the HaCat cell line, indicating a potential prospect for dental health product.

## INTRODUCTION

Dental caries and periodontal disease is an infection in the mouth with high prevalence (Merchant 2012). Both of these diseases occur in nearly 95% of all diseases in the oral cavity (Çaglar *et al*. 2005). Caries is considered an endogenous disease because the major bacterial species that induces the demineralisation is *Streptococcus mutans*. Another virulence property of *S. mutans* related to pathogenicity caries is the formation of the enzyme glucosyltransferase (GTF), which plays a role in the origin of food fermenting saccharides and has an impact on the decrease in salivary pH (Ribeiro *et al*. 2012). Dental biofilm, or dental plaque, formed by *S. mutans* is the cause of dental caries. The ability to form biofilm in this species causing caries, an expression of virulence properties of *S. mutans* (Bowen 1996). This conversion explains that *S. mutans* successfully forms biofilm mass on the enamel surface, which is the initial cause of dental caries (Fejerskov 2004).

Upon invasion by pathogenic bacteria, bacterial antigens (molecules on the surfaces of bacteria) can activate macrophages, which lead to the secretion of pro-inflammatory cytokines, such as tumour necrosis factor (TNF)-α, interleukin (IL)-1β, and IL-8 (Wang *et al*. 2000). Both Gram-positive and Gram-negative bacteria can modulate this immune response in slightly similar mechanisms. Peptidoglycan (major cell wall component of Gram-positive bacteria, and also exists in the cell wall of Gram-negative bacteria as a thin layer) has been considered as a pro-inflammatory cytokines modulator in the previous study (Wang *et al*. 2000).

Periodontal disease is characterised by increasing pathogenicity or virulence of pathogenic microorganisms, increased inflammation, and markers levels of the inflammation processes. One of the oral bacteria species that are often reported to be associated with the pathogenicity of periodontal disease, especially chronic periodontitis is *Porphyromonas gingivalis* (*P. gingivalis*) (How *et al*. 2016). Unlike dental caries that involves the role of *S. mutans*, the periodontitis mechanism, besides involving the role of *P. gingivalis* and a number of other oral bacteria species, is also determined by the response of the host cell, in particular the inflammatory response of periodontal tissue destruction such as gingivitis. LPS from *P. gingivalis* will increase the production of cytokines such as interleukin (IL)-1, IL-6, IL-8, and tumor necrosis factor (TNF)-α in gingival fibroblasts as a pro-inflammatory mediator expressed by epithelial cells (Wang & Ohura 2002).

Epithelium contains keratinised cells (keratinocytes), and keratinocytes can be found in most type of oral epithelial tissues (Bragulla & Homberger 2009), including gingival epithelial cells, which has been used to study oral diseases such as dental caries and periodontitis (Wilson 2013). Keratinocytes have both innate and adaptive immune systems, which include the expression of IL-8 as the prominent pro-inflammatory cytokines and antimicrobial peptides, called human beta-defensins (hBD) (Wilson 2013; Jiang *et al*. 2012). If the epithelial cells are exposed to periodontal disease-causing bacteria, such as *Aggregatibacter actinomycetemcomitans* and *P. gingivalis*, it will produce human beta defensin-2 as a defense mechanism of the body (Tribble & Lamont 2010).

Bacteriotherapy, or the use of harmless bacteria to displace pathogenic microorganisms, has been proven effective in controlling a wide variety of infectious diseases. A therapy using probiotic is one technique that can be used as a therapy against microorganisms. Probiotics introduced by the International Scientific Association for Probiotics. Probiotics are living microorganisms that are beneficial to the body in the appropriate amount of bacteria. Certain studies have shown that probiotic cultures may improve oral health (Stamatova *et al*. 2007). *Lactobacillus reuteri* is one species of probiotic bacteria that has many benefits on health because it can inhibit the growth of bacteria that cause periodontal disease in the oral cavity (Sinkiewicz 2010). *L. reuteri* is considered a heterofermentative obligate bacteria that can be found in the digestive tract and can produce reuterin, a protein with broad-spectrum antibiotic properties and is effective in a wide pH range and resistant to proteolytic and lipolytic enzymes (El-Ziney & Debevere 1998).

Oral probiotic is expected to survive in the oral ecosystem conditions. Several studies have shown that consumption of probiotics only lasted for two weeks after ingestion. Normal oral bacteria that has been tried to be used as probiotic are *Lactococcus lactis, Lactobacillus acidophilus, Streptococcus thermophilus, Streptococcus mutans*, and *Streptococcus salivarius* (Burton *et al*. 2005). *L. reuteri* is regarded as normal gastrointestinal flora (El-Ziney & Debevere 1998), however, some studies claim that these bacteria are also present in the oral cavity as normal flora (Iniesta *et al*. 2012).

In the pprevious study, Sinkiewicz has proved an overall decrease in gingival bleeding, plaque formation, and other symptoms that usually arise in the case of moderate to severe gingivitis in patients after consumption chewing gum containing *L. reuteri* (Sinkiewicz 2010). Previous *in vivo* research showed that probiotic *L. reuteri* (Prodentis) is able to reduce colonies number of *Aggregatibacter actinimycetemcomitans, Prevotella intermedia* and *P. gingivalis*in saliva (Vivekananda *et al*. 2010). The protective mechanism of the barriers against the growth of bacteria that cause dental caries and periodontal disease by *L. reuteri* is through reuterin, the antimicrobial substance produced by *L. reuteri* (Saha *et al*. 2014).

Probiotic bacteria *L. reuteri* is known to produce an antimicrobial and anti-inflammatory compound called reuterin. However, the reuterin isolate probiotic effect of clinical strains of *L. reuteri*, particularly on interleukin and human beta-defensin, has not been further researched. Taking this research gap into consideration, in the present study, we attempted to discover clinical strains of reuterin isolate *L. reuteri* which possess probiotic properties as an anti-inflammatory agent. This study aims to investigate the ability of reuterin isolate from *L. reuteri* Indonesian strain in reducing mRNA expression of interleukin-8 (IL-8) and increasing mRNA expression of human beta-defensin (HBD)-2 gene produced by epithelial cell after exposure to oral bacteria.

## MATERIALS AND METHODS

### Bacterial Culture (*S. mutans, P. gingivalis* and *L. reuteri*)

The bacterial cultures were obtained by following the standard protocols at Oral Biology Laboratory, Faculty of Dentistry, University of Indonesia, Jakarta. Bacterial strains of *Streptococcus mutans* ATCC-25175 and *Porphyromonas gingivalis* ATCC-33277 were maintained in stock cultures frozen at −80°C in brain heart infusion (BHI) broth containing 20% (v/v) glycerol (Biomatik, Wilmington, Delaware, USA). *S. mutans* ATCC-25175 was cultured in brain heart infusion (BHI) broth (Thermo Fisher Scientific, Waltham, Massachusetts, USA) and incubated in anaerobic conditions with CO_2_ supply at 37°C. *P. gingivalis* ATCC-33277 was cultured in BHI broth and incubated in a GasPak jar system (Becton Dickinson, Franklin Lakes, NJ, USA). *L. reuteri* ATCC-55730 was cultured in De Man, Rogosa, and Sharpe (MRS) broth (Thermo Fisher Scientific, Waltham, Massachusetts, USA) and incubated in anaerobic conditions at 37°C. Prior exposing to HaCat cells, *S. mutans* and *P. gingivalis* were killed by heating both bacteria at 80°C for 30 min.

### L. reuteri isolation from the clinical sample

This experiment has been approved by the Ethics and Research Committee of the Faculty of Dentistry, Trisakti University, Jakarta, Indonesia, under process number 118/KE/FKG/12/2014. *L. reuteri* isolated from Indonesian’s saliva, and species was confirmed by PCR, using 16S rRNA specific gene from our previous study (Widyarman *et al*. 2018b). Primers for 16S rRNA for gene amplification were 5′ ACC TGA TTG ACG ATG GAT CAC CAGT (forward); CCA CCT TCC TCC GGT TTG TCA 3′ (reverse) (First Base, Singapore Science Park, Queenstown, Singapore) (Kwon *et al*. 2004).

### Reuterin Isolation

*L. reuteri* was cultured in MRS broth, maintained overnight in anaerobic conditions at 37°C. Its cells were then harvested by centrifugation at 5,000 × g for 15 min at 20°C, washed with PBS (pH 7.4) (Biomatik, Wilmington, Delaware, USA), and centrifuged for the second time at 5,000 × g for 15 min. The cells were resuspended at a concentration of 1.5 × 10^10^ CFU/mL in 300 mM glycerol solution, then incubated at 37°C for 3 h in anaerobic conditions. After incubation, the suspensions were centrifuged at 5,000 × g for 15 min (the living bacteria as pellets), and the supernatants were filtered through 0.22 μm pore-sized-membrane filters (Merck, Darmstadt, Germany). The filtered supernatants were then measured using Bradford assay and resulted that the reuterin concentration was 208.06 μg/mL (Widyarman & Theodorea 2021).

### HaCat Cells Culture

The HaCat cells were washed by addition of 7 mL PBS and centrifuged at 1000 × g for 10 min. The supernatant was discarded, and 3 mL of the medium (containing DMEM, 10% (v/v) fetal bovine serum (FBS), 1% (v/v) fungizone as an antifungal agent, and 1% (v/v) penicillin-streptomycin (VWR Life Science, Radnor, Pennsylvania, USA) was added into the pellets. Then, a 25 cm^2^ flask culture dish (Biologix, Lenexa, Kansas, USA) containing 10 mL of DMEM was prepared, and the cells were added to the flask. The flask was put into an incubator and was maintained in anaerobic conditions (5% CO_2_) at 37°C, with 96% relative humidity for three days.

### MTT (3-[4,5-dimethylthiazol-2-yl]-2,5diphenyltetrazolium bromide) Assay

To study the cytotoxicity effects of reuterin on HaCat cells, an MTT assay was performed. The cells were aliquoted into a flat-bottom (Biologix, Lenexa, Kansas, USA), 96-well TC plate (10^4^–10^6^ cells/mL) in 200 μL DMEM solutions and reuterin was added into each well with concentrations ranging from100%, 50%, 25%, 12.5% and 6.25%. The incubation time was set for 15 min, 30 min, 1 h, 3 h, 6 h, and 24 h, subsequently at 37°C. Experiments were done in triplicate and repeated two times with different incubation times. After that, 100 μL of MTT reagent (VWR Life Science, Radnor, Pennsylvania, USA) (5 mg MTT in 1 mL 0.9% NaCl) was added into each well. The well was then incubated for 3 h at 37°C with 5% CO_2_ supplementation. After that, 100 μL of acidified isopropanol was added into each well as a stopper for the reaction and incubated again for 1 h in a shaker. Optical density (OD) of HaCat cells was measured using an ELISA reader (SAFAS, Quai Antoine 1er, Monaco) at 490 nm.

### Harvesting HaCat Cells and Exposure to Oral Pathogens

To harvest HaCat cells, the cells in the flask were washed using 2 mL of PBS three times, followed by the addition of 1.5 mL of trypsin/EDTA into each flask. The flask was then incubated for 10 min. An inverted microscope (Zeiss, Oberkochen, Germany) was used to observe the dissociation of the cells. After that, 4 mL of DMEM was added into each flask, and the suspensions were transferred into a new 15 mL tube, followed by centrifugation at 1000 × g for 10 min. Next, the supernatant was discarded, and the pellet was resuspended by adding 1 mL of DMEM. The number of living cells was counted using a flow cytometer. The cells were transferred into a 24-well plate (cell density approximately 1 × 10^5^/mL), and the plate was incubated for 24 h, 37°C in anaerobic conditions (5% CO_2_). After that, *S. mutans* and *P. gingivalis* (10^7^ CFU/mL of quantities) were separately added to each well. The plate was further incubated for an overnight incubation period at 37°C in 5% CO_2_ to induce inflammatory responses of HaCat cells. Various concentrations of reuterin (100%, 50%, 25% and 12.5 %) were added into each well following the design, according to different incubation times: 15 min, 3 h and 24 h at 37°C and 5% CO_2_. The medium was then discarded, while the cells were washed using PBS.

### RNA Extraction

RNA extraction was performed using TRIzol reagents (Invitrogen/Thermo Fisher Scientific, Waltham, Massachusetts, USA). Approximately 1 mL of TRIzol was added into the cells, followed by incubation for 5 min at room temperature. Then, 200 μL of chloroform was added. Next, the suspension was transferred into a new 1.5 mL microtube, and the tube was flipped for 15 sec to homogenise the mixture. After that, the suspension was incubated for 3 min at room temperature and then was centrifuged at 12,000 × g for 15 min at 4°C. The upper layer (aqueous phase) was taken out and then transferred into a new 1.5 mL microtube. 100% isopropanol at an amount of 0.5 mL was added into the tube and then incubated at room temperature for 10 min. The tube was then centrifuged at 12,000 × g for 10 min at 4°C. The supernatant was discarded, and 75% ethanol at the amount of 1 mL was added into the tube. The mixture was homogenised using a vortex mixer and then was centrifuged again at 7,500 × g for 5 min at 4°C. The supernatant was discarded, while the pellet was air-dried for 10 min at room temperature. The RNA-containing pellet was then resuspended again by adding 20 μL of ddH_2_O, and incubated in a thermoblock machine (Biosan, Riga, Latvia) at 57°C for 15 min. After that, the pellet was stored at −70°C. A spectrophotometer was used to determine RNA concentration. The isolated RNA was taken out (approximately 2 μL) and diluted in 498 μL ddH_2_O. Then, RNA solution was fed into a glass cuvette, and the cuvette was inserted into the spectrophotometer.

### cDNA Synthesis

After the concentration of RNA was measured, reverse transcriptase (RT)-PCR was performed to obtain cDNA, with Thermo Fisher Scientific’s GeneAmp Gold RNA PCR Reagen Kit using random oligo (dT) primer and 1 μg of the RNA template, following The Two-Step RNA PCR Reaction with the total volume of 25 μL. The hybridisation was set at 25°C for 10 min, followed by reverse transcription at 42°C for 12 min. The reagent activation was set at 95°C for 10 min, followed by denaturation at 94°C for 20 sec, annealing at 62°C for 1 min, and final extension at 72°C for 7 min with 43 PCR cycles (Thermo Fisher Scientific, Waltham, Massachusetts, USA).

### Real-time Quantitative (qRT)-PCR

The final part of the study was to evaluate the effect of reuterin on inflammatory responses of pathogens-induced HaCat cells by measuring the expression of interleukin (IL)-8 and human beta-defensin (HBD)-2 using the Real-Time quantitive PCR method. Specific primers were used for GAPDH as a housekeeping gene, IL-8, and HBD-2 (the sequence of each primer was shown in Table 1). The PCR compositions were: 5 μL of 2x SYBR green, 0.5 μL forward primers (10 mM), 0.5 μL reverse primers (10 mM), 5 μL of cDNA sequence, and nuclease-free water up to 20 μL of total volume. A StepOne Plus V2.3 Real-Time Quantitative PCR machine (Applied Biosystem, Foster City, California, USA) was used in this research to obtain comparative CT (ΔΔCT) from each target. RT PCR was performed in duplicate.

### Statistical Analysis

One-way ANOVA test was applied to reveal significant differences of IL-8 and hBD-2 mRNA expressions in HaCat cells exposed with *S. mutans, P. gingivalis* and reuterin isolated from *L. reuteri* in different concentration and incubation times. Differences were considered statistically significant if *p* < 0.05. Statistical calculations were performed with SPSS Statistics for Windows software version 20 (IBM, New York, USA).

## RESULTS

### MTT Assay

**Figure 1:**
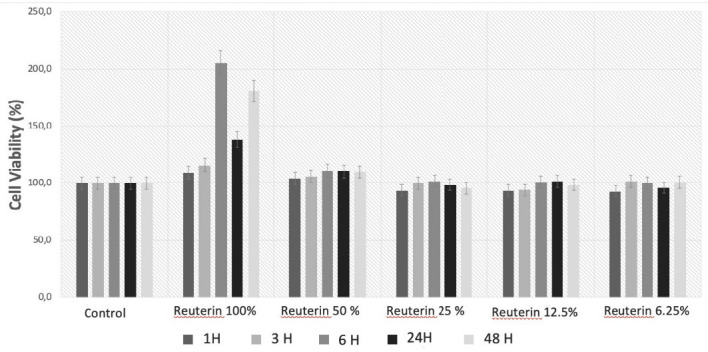
Viability of HaCat cells after exposure to reuterin with the concentration of 100%, 50%, 25%, 12.5% and 6.25%.

According to ISO 10993-5:2009, the percentage of cell viability above 80% is considered safe with no cytotoxicity properties to living cells, between 80%–60% of low toxicity, medium toxicity is 60%–40%, and below 40% is considered as toxic. The lowest cell viability in this research was 93%, which suggested that reuterin isolated from *L. reuteri* BEA-230424 did not possess cytotoxicity against HaCat cells (Fig. 1).

**Figure 2:**
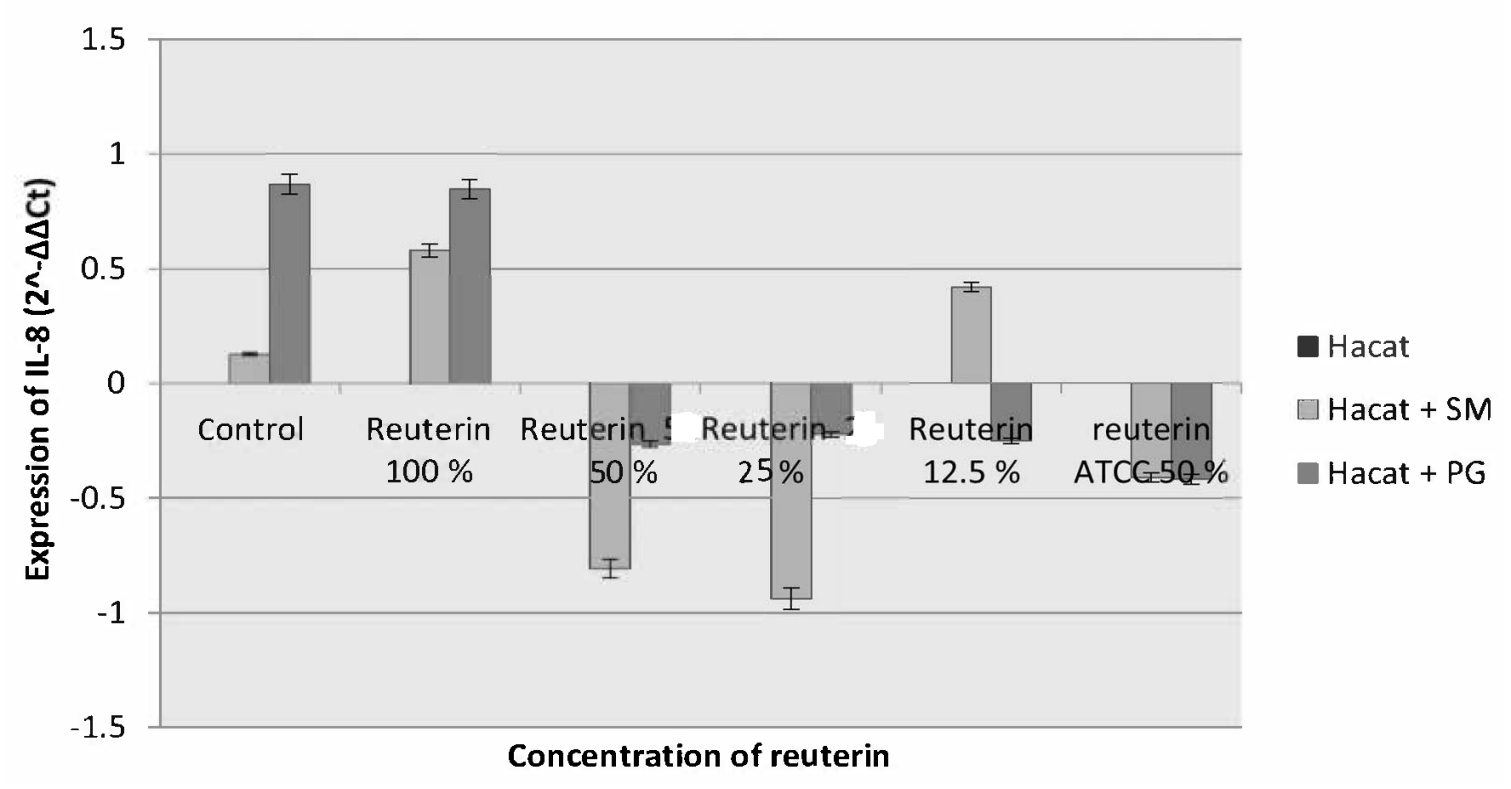
IL-8 expression of HaCat cells after exposure to *S. mutans* and *P. gingivalis* and given reuterin with 1 h incubation period.

The 1 h incubation period suggested a reduction in the expression of IL-8 in HaCat cells induced by *S. mutans* on reuterin of concentration of 50%, 25% and reuterin ATCC, whereas in HaCat cells induced by *P. gingivalis* there was the reduction in the expression of IL-8 in all given concentrations of reuterin, except on reuterin concentration of 100% (Fig. 2).

**Figure 3:**
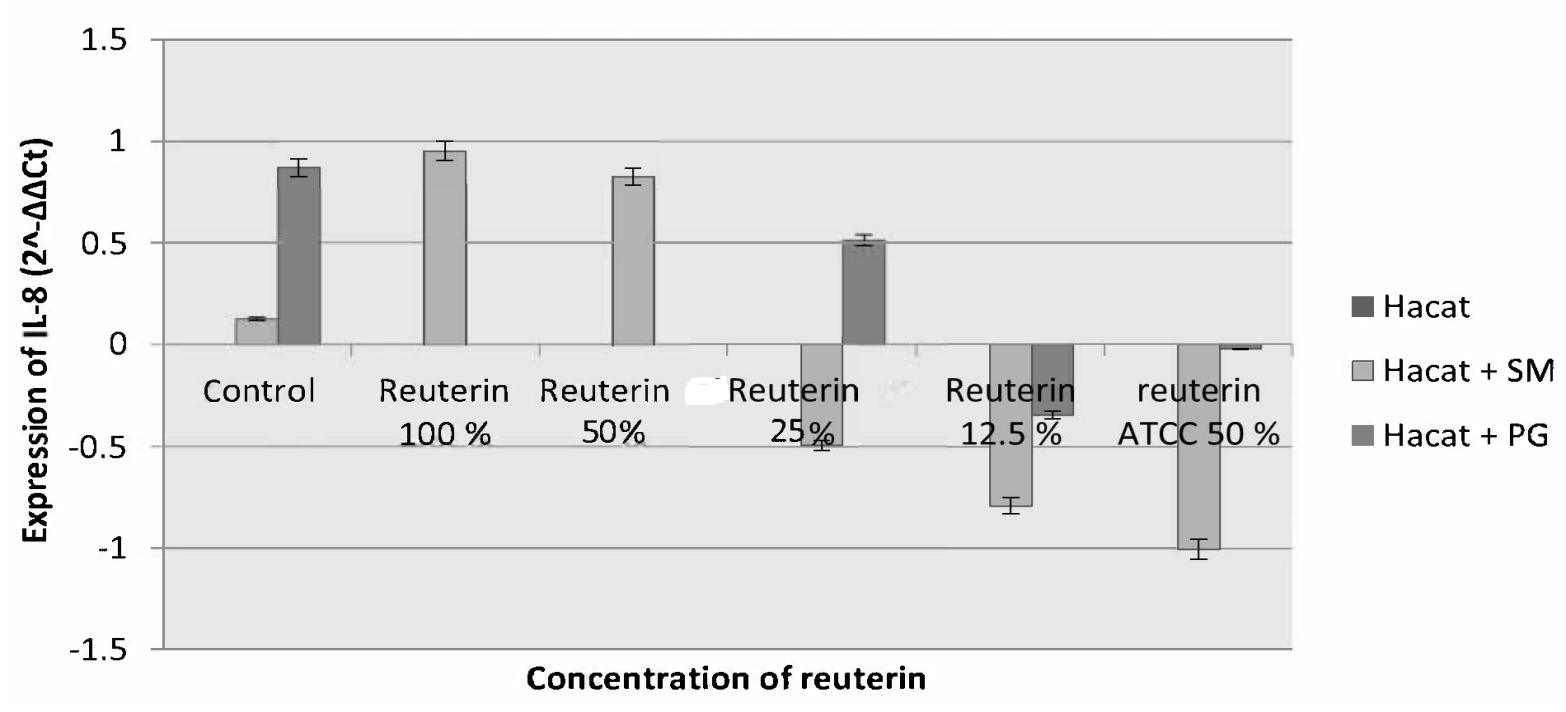
IL-8 expression of HaCat cells after exposure to *S. mutans* and *P. gingivalis* and given reuterin with the 3 h incubation period.

The 3 h incubation period resulted in a reduction of IL-8 expression in HaCat cells induced by *S. mutans* with reuterin concentration of 25%, 12.5%, and reuterin ATCC-55730, and there was an increment in the concentration expression of 100% and 50%, while on the HaCat cells exposed to *P. gingivalis* there was a reduction occurred in the expression of IL-8 regarding all reuterin concentrations (Fig. 3).

**Figure 4:**
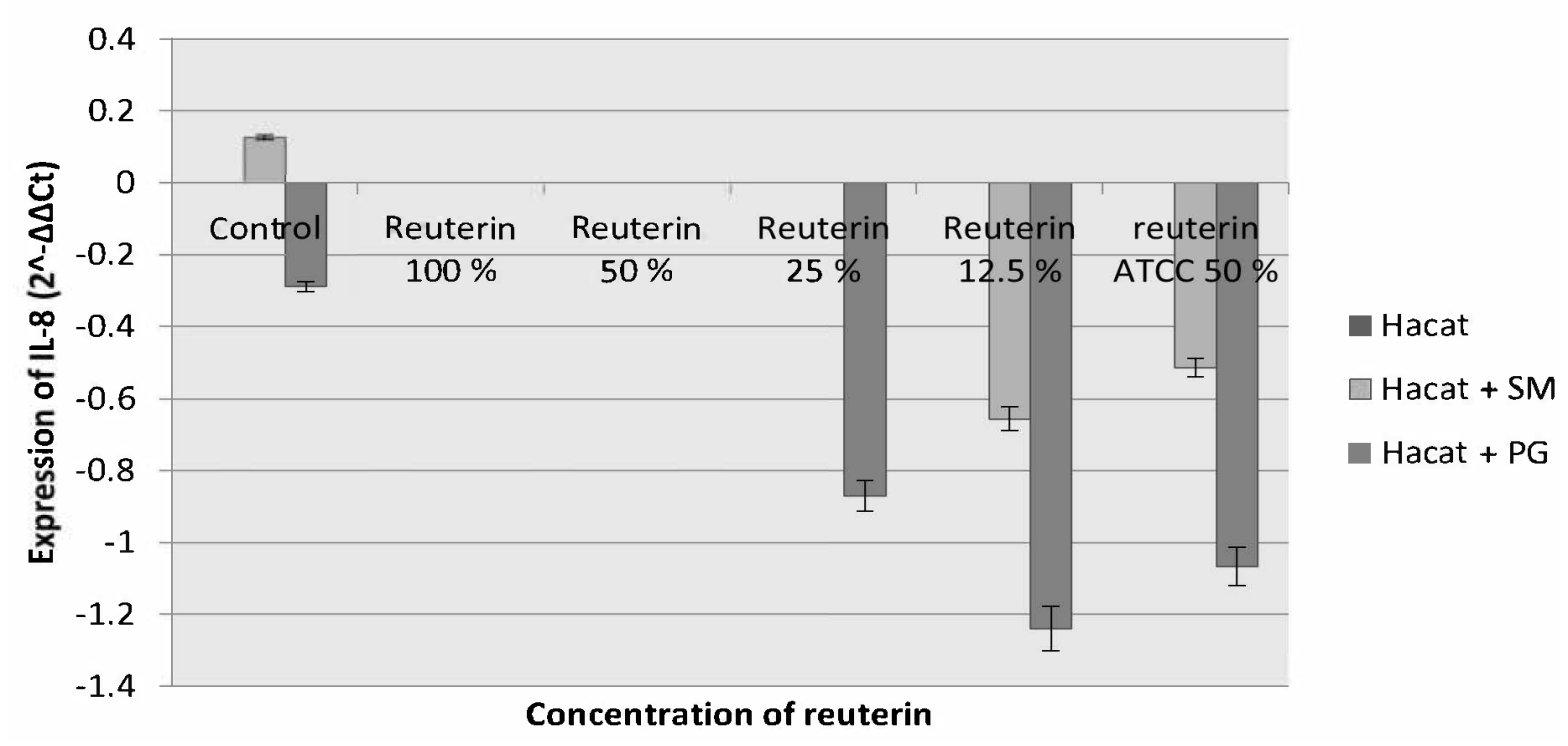
IL-8 expression of HaCat cells after exposure to *S. mutans* and *P. gingivalis* and given reuterin with the 24 h incubation period.

In the 24 h incubation period, the reduction of IL-8 expression in HaCat cells induced by *S. mutans* and *P. gingivalis* became evident on all reuterin concentrations. Therefore, it can be assumed that the most optimum incubation period for reuterin to reduce the inflammatory response was 24 h, with reuterin concentration of 12.5% (Fig. 4).

**Figure 5:**
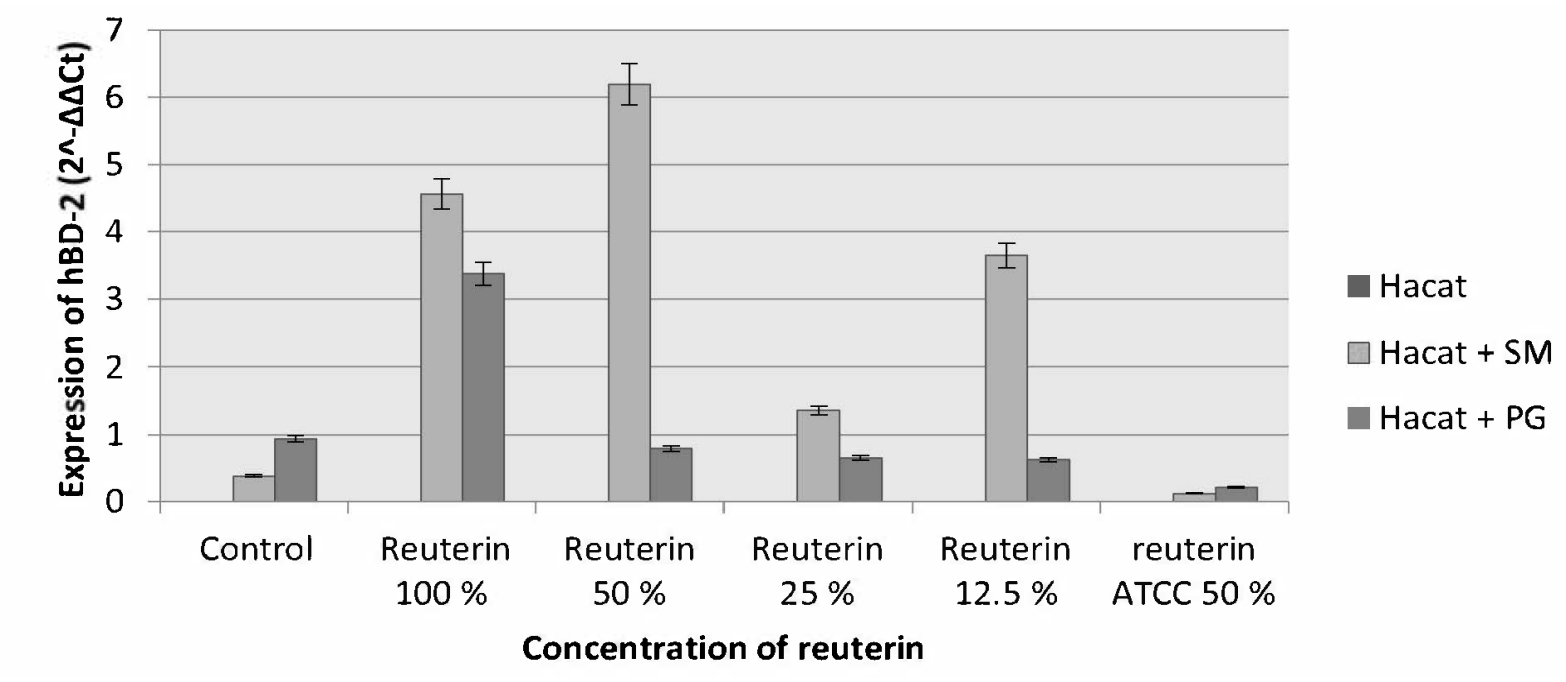
HBD-2 expression of HaCat cells after exposure to *S. mutans* and *P. gingivalis* and given reuterin with the 1 h incubation period.

**Figure 6:**
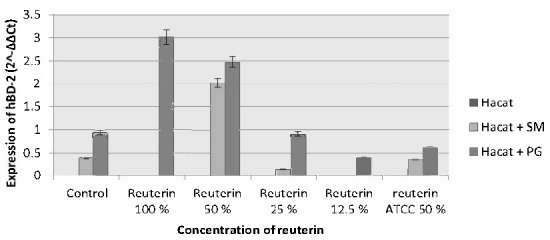
HBD-2 expression of HaCat cells after exposure to *S. mutans* and *P. gingivalis* and given reuterin with the 3 h incubation period.

In 1 h incubation period, an increment in HBD-2 expression of HaCat cells induced by *S. mutans* and *P. gingivalis* on all reuterin concentrations can be seen, and the biggest increment in HBD-2 expression was with reuterin at a concentration of 50% and 100% (Fig. 5). In the 3 h incubation period, the increment of HBD-2 expression in HaCat cells induced by *S. mutans* occurred only on reuterin concentration of 50%, while on *P. gingivalis*-induced HaCat cells, there was an increase in HBD-2 expression on all reuterin concentrations, except for 12.5% reuterin where there was a slight decrement (Fig. 6).

**Figure 7:**
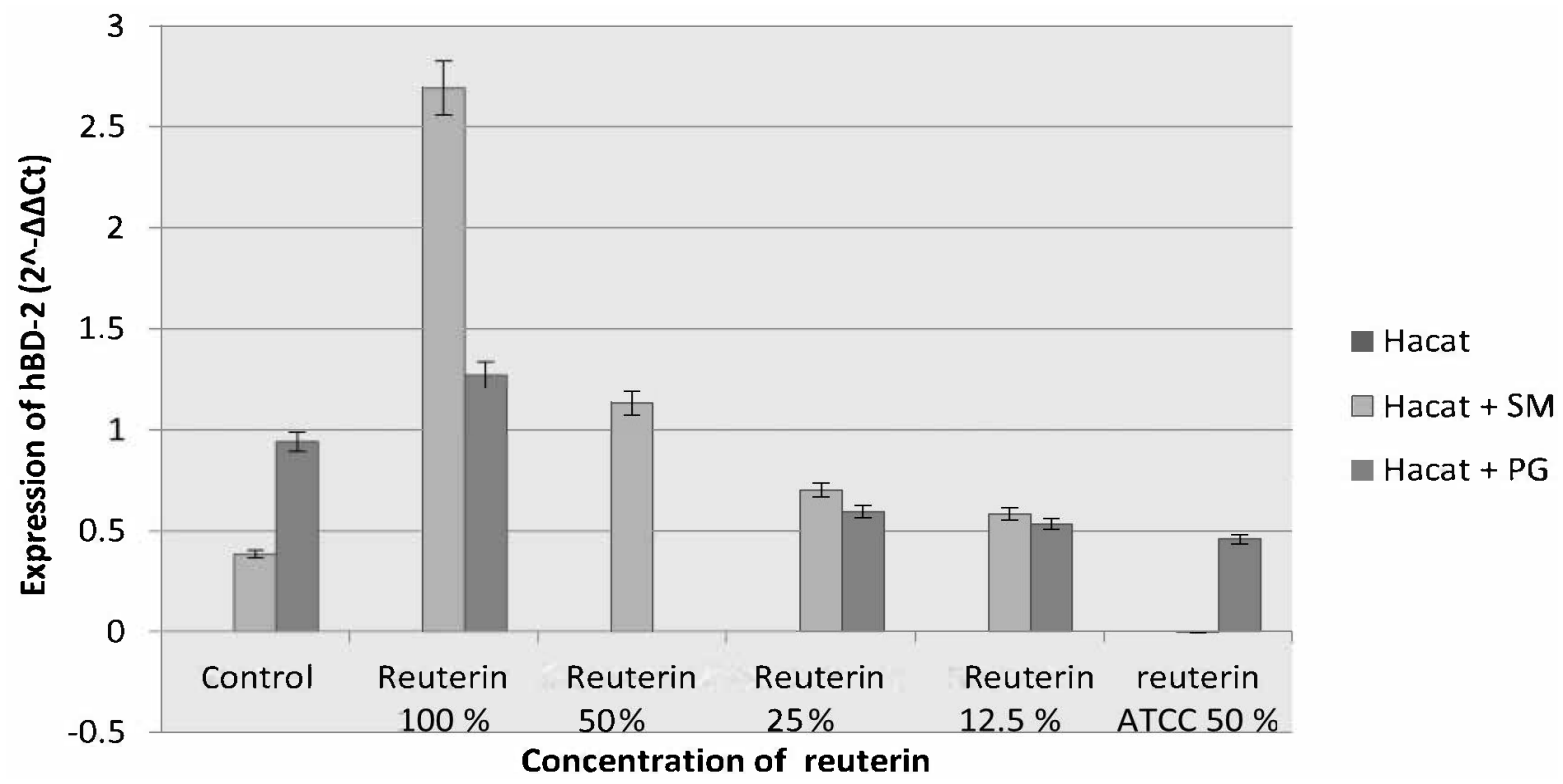
HBD-2 expression of HaCat cells after exposure to *S. mutans* and *P. gingivalis* and given reuterin with the 24 h incubation period.

After 24 h, an increment of HBD-2 expression on HaCat cells induced by *S. mutans* and *P. gingivalis* was found on all reuterin concentrations, with the biggest increment of HBD-2 expression was with 100% reuterin. Therefore, it can be assumed that the best incubation period for reuterin to increase the expression of HBD-2 was 24 h. The higher reuterin concentration, the greater HBD-2 expressed by epithelial cells (Fig. 7).

## DISCUSSION

This research studied the effect of reuterin on human epithelial cells that have been in induced by *S. mutans* as Gram-positive bacteria and *P. gingivalis* as Gram-negative bacteria. The dental plaque accumulation at the gingival margin containing lipoteichoic acids (LTA) derived from the cell wall of Gram-positive bacteria, while the subgingival plaque associated with the periodontal disease increased the number of lipopolysaccharides (LPS) from Gram-negative bacteria (Pöllänen *et al*. 2000). In the oral cavity, these bacteria will form biofilms and accumulate in the biofilms, which can induce a response in the oral mucosal epithelial cells. *S. mutans* as pathogenic bacteria can stimulate the formation of pro-inflammatory cytokines. Glucosyltransferase enzyme (GTFs) of *S. mutans*, both GtfC and GtfD can induce the production of interleukin (IL)-6 in the cells (Chia *et al*. 2002). Furthermore, epithelial cells will express antimicrobial peptides such as ribonuclease 7 (RNase-7), and psoriasin (PSO), as well as pro-inflammatory cytokine mediators such as interleukin-8 (IL-8) and 5-lipoxygenase (5-LO) (Eberhard *et al*. 2009).

Reuterin produced by *L. reuteri* has decreased the expression of IL-8. On the other hand, the activity of reuterin against HBD-2 expression on the transcriptional level was much different from IL-8. The higher reuterin concentration, the greater HBD-2 expressed by the cells. Commensal probiotic bacteria are such good inducers for beta defensin-2 expression in oral cavity epithelial cells (Wallace *et al*. 2011). Previous studies using murine parotid gland epithelial cells have found that *L. reuteri* induction to epithelial cells can induce beta defensin-2 and reduce the number of bacterial populations related to dental caries (Kusumaningsih *et al*. 2016). Cellular responses against pathogenic microorganisms vary depending on the specific characteristic of pathogens, such as products, concentration, and duration of exposure (Feezor *et al*. 2003). This research showed the potential difference of IL-8 and HBD-2 expression in HaCat cells prior to exposure to both Gram-positive and Gram-negative bacteria.

In this study, after 1 h of reuterin 100% concentration addition, the level of IL-8 mRNA expression induced with *S. mutans* and *P. gingivalis* was not reduced. This was correlated with the hBD-2 innate immune response against the resulting IL-8 mRNA expression, which the hBD-2 mRNA expression was has highly increased with this high concentration of reuterin at this incubation period. Furthermore, after incubation with reuterin for 24 h, both *S. mutans*- and *P. gingivalis*-induced IL-8 mRNA expressions were significantly reduced, compared with the first 1 h and 3 h of incubation. These results were considered beneficial to the epithelial cells since prolonged production of IL-8 can cause cell destruction due to the accumulation of neutrophils (Cao *et al*. 2005).

*In vivo* research showed that *Lactobacillus* probiotics can modulate both pro- and anti-inflammatory responses (Gourbeyre *et al*. 2011). *L. reuteri* has the ability to suppress pro-inflammatory cytokines, such as TNF-α, by converting L-histidine into histamine, an immunoregulatory signal. Histamine can later suppress the activation of mitogen-activated protein (MAP)-kinase signaling pathway, thus resulting in a delay in cytokines production via histamine receptor type 2 (H2) on cell hosts (Thomas *et al*. 2012). *L. reuteri* 6475 can inhibit TNF-α, a pro-inflammatory cytokine, in monocyte-derived macrophages isolated from children with Crohn’s disease, as well as toll-like receptor (TLR) 2 and TLR4-activated human and murinemonocytoid cell lines (Lin *et al*. 2008).

*L. reuteri* in the form of planktonic cells and biofilms can produce reuterin, although the amount of reuterin produced could be different depending on bacterial strain (Jones & Versalovic 2009). In the presence of glycerol, *L. reuteri* can synthesise 3-hydroxypropionaldehyde (3-HPA). This molecule of 3-HPA was secreted into the medium simultaneously with the formation of hydrates and dimers, forming an equilibrium dynamic multi-component called reuterin (Vollenweider & Lacroix 2004). Previous research has also demonstrated that *in vitro* studies in intestinal epithelial cells demonstrated that *L. reuteri* strains have intrinsic pro-inflammatory activity in cultured cells, but the strains differentially inhibit LPS-induced IL-8 production (Liu *et al*. 2010). *L. reuteri* requires the presence of glycerol over a certain period to produce reuterin to develop the beneficial capability of inhibiting oral pathogenic bacteria and acting as an anti-inflammatory agent to reduce inflammatory mediators, such as IL-8 and HBD-2 (Widyarman *et al*. 2018a).

In a previous study focused on probiotic’s effect against LPS-induced IL-8 production using intestinal epithelial cells LPS derived from Gram-negative bacteria binds to signaling proteins, such as CD14, thus leading to activation of nuclear factor (NF)-κB, a central regulator of innate immune systems (Versalovic *et al*. 2008). Activation of NF-κB can result in the production of pro-inflammatory chemokines, including IL-8 (Savkovic *et al*. 1997; Cario *et al*. 2000; Funda *et al*. 2001). Human-derived *L. reuteri* strains have significantly suppressed IL-8 production by inhibiting NF-κB activation at a terminal step in the related pathways (Savkovic *et al*. 1997). *L. reuteri* and bacteriocin (reuterin) secreted by each strain may also potentially inactivate NF-κB at the transcriptional level, related to *Lactobacillus reuteri*-specific immunoregulatory (*rsiR*) gene, which can modulate pro-inflammatory cytokines production (Hemarajata *et al*. 2013).

## CONCLUSIONS

Reuterin may inhibit inflammatory response signified by reduction of IL-8 expression and increases the expression of human beta-defensin 2 by HaCat cells. Reuterin has no toxicity to epithelial cells and also has the ability to influence the inflammatory response by increasing the expression of HBD-2, which works as a peptide antibiotic. With the ability of this substance as an anti-inflammatory, therefore, the substance from this probiotic bacterium is considered as an anti-inflammatory ingredient. However, further research is needed to confirm this effect *in vivo*.

## Figures and Tables

**Table 1:** Primers for IL-8, hBD-2 and GAPDH gene

Primers	Sequence (5′ – 3′)
IL-8 Forward	TCT CTT GGC AGC CTT CCT
IL-8 Reverse	ACT GAA CCT GAC CGT ACA TGT CTT TAT GCA CTG ACA TCT
hBD-2 Forward	GGT GTT TTT GGT GGT ATA GGC
hBD-2 Reverse	AGG GCA AAA GAC TGG ATG ACA
GAPDH Forward	CTG AGT ACG TCG TGG AGT C
GAPDH Reverse	ACT GAA CCT GAC CGT ACA CAG AGA TGA TGA CCC TTT TG
